# Comparison of the standard and boosted sterile insect techniques for the suppression of *Aedes albopictus* populations under semi-field conditions

**DOI:** 10.1051/parasite/2025047

**Published:** 2025-08-27

**Authors:** Marlène Dupraz, Renaud Lancelot, Gorgui Diouf, Marco Malfacini, Lucie Marquereau, Louis-Clément Gouagna, Marie Rossignol, Fabrice Chandre, Thierry Baldet, Jérémy Bouyer

**Affiliations:** 1 UMR ASTRE, CIRAD-INRAE-Univ. Montpellier Montpellier 34398 France; 2 UMR ASTRE, CIRAD-INRAE-Univ. Montpellier, Plateforme CYROI Sainte Clotilde 97491 La Réunion France; 3 Laboratoire National d’Elevage et de Recherches Vétérinaires, ISRA BP 2057 Dakar Sénégal; 4 Centro Agricoltura Ambiente “G. Nicoli” 40014 Crevalcore Italy; 5 UMR Mivegec, IRD-CNRS-Univ. Montpellier Saint-Pierre 97410 La Réunion France; 6 UMR Mivegec, IRD-CNRS-Univ. Montpellier Montpellier 34394 France

**Keywords:** Pyriproxyfen, Biocide vectorization, Competitiveness, *Aedes albopictus*, SIT, Mosquito control

## Abstract

Innovative control tools are needed against *Aedes* mosquitoes. The boosted sterile insect technique (bSIT) consists of treating sterile males with a biocide prior to their release to contaminate larval habitats. We compared the efficacy of SIT and boosted SIT to prevent the emergence of adult *Aedes albopictus* in large cages. We tested two sterile-to-fertile male ratios: 5:1 (SIT5) and 1:1 (SIT1), with and without pyriproxyfen enhancement (bSIT or SIT). The eggs were collected in ovitraps and the immature stages were monitored until adult emergence or up to 15 days after hatching to estimate the relative risk (*RR*) of adult emergence compared to the control category. The concentration of pyriproxyfen in the ovitrap water did not change when sterile males were released with females or alone (*χ*^2^ = 0.99, *df* = 1, *p* = 0.547). This concentration was higher when the sterile-to-fertile male ratio was increased from 1:1 to 5:1: *χ*^2^ = 18.8, *df* = 1, *p* = 0.006. All four treatment categories were effective in suppressing mosquito populations. With a relative risk *RR* = 0.194 95% CI [0.128; 0.275], SIT5 was the most effective. Boosted SIT was not as effective as SIT. However, bSIT1 (*RR* = 0.418 [0.351; 0.492]) and bSIT5 (*RR* = 0.512 [0.431; 0.596]) were equally effective. Boosted males directly vectored pyriproxyfen to breeding sites. Boosted SIT was more effective than SIT alone with a low sterile-to-fertile male ratio. Under operational conditions, it could be initially deployed to suppress the target population and then switched to standard SIT.

## Abstract (fr)

Des outils innovants sont nécessaires pour lutter contre les moustiques *Aedes*. La technique de l’insecte stérile renforcée (TIS-R) consiste à traiter les mâles stériles avec un biocide avant leur lâcher afin de contaminer les gîtes larvaires. Nous avons comparé l’efficacité de la TIS et de la TIS-R pour prévenir l’émergence d’*Aedes albopictus* adultes dans de grandes cages. Nous avons utilisé deux ratios entre mâles stériles et fertiles : 5:1 (TIS5) ou 1:1 (TIS1), traités ou non par du pyriproxyfène (TIS-R5 et TIS-R1). Les œufs ont été collectés dans des pièges à oviposition et les stades immatures ont été surveillés jusqu’à l’émergence des adultes ou jusqu’à 15 jours après l’éclosion afin d’estimer le risque relatif (*RR*) d’émergence des adultes par rapport à la catégorie témoin. La concentration de pyriproxyfène dans l’eau des pièges à oviposition n’a pas changé lorsque les mâles stériles étaient lâchés avec les femelles, ou seuls (*χ*^2^ = 0,99, *df* = 1, *p* = 0,547). Cette concentration était plus élevée lorsque le rapport entre mâles stériles et fertiles passait de 1 à 5 : *χ*^2^ = 18.8, *df* = 1, *p* = 0,006. Les quatre traitements ont permis de supprimer les populations de moustiques. La TIS-R5 s'est avérée la plus efficace : *RR* = 0,194 [0,128; 0,275]. La TIS-R n’était pas aussi efficace que la TIS. Cependant, la TIS-R1 (*RR* = 0,418 [0,351; 0,492]) et la TIS-R5 (*RR* = 0,512 [0,431; 0,596]) étaient d’efficacité similaire. Les mâles traités ont directement transmis le pyriproxyfène aux gîtes larvaires. La TIS renforcée s’est avérée plus efficace que la TIS standard avec un ratio entre mâles stériles et fertiles peu élevé. En conditions opérationnelles, elle pourrait être utilisée en début de lutte pour supprimer la population cible, puis remplacée par la TIS standard.

## Introduction

*Aedes* mosquitoes are vectors of human pathogens such as yellow fever, dengue, chikungunya, and Zika viruses. Due to many factors, including human mobility and environmental and climate change, these *Aedes*-borne viral diseases are increasing in tropical and subtropical regions. In Europe, climate change is leading to higher temperatures and unexpected weather conditions, which favor the proliferation of mosquitoes, the multiplication of viruses in their insect vectors, and the transmission of associated mosquito-borne infections even during the winter period in temperate areas [1, 38]. In fact, local transmission/outbreaks of dengue virus have increased in mainland Europe over the last ten years [20]. The global economic cost of *Aedes aegypti* and *Aedes albopictus* mosquitoes and related diseases was estimated to be USD 94.7 billion over 45 years (1975–2020), and more than USD 300 billion when calculating the after-effects (such as osteoarthritis for chikungunya) and long-term costs [50]. Thus, a 14-fold cost increase was estimated since the emergence of the Zika and chikungunya viruses around the world. Meanwhile, vector control lacks resources, particularly large-scale financial support, which favors the proliferation of *Aedes* mosquitoes and the transmission of *Aedes*-borne viruses. Although individual protective measures are available, collective methods, such as systematic and extensive destruction of breeding sites, are the most effective preventive measures. However, current methods of controlling mosquito populations have limitations [57]. Insecticides, although effective in the short term, are costly and harmful to the environment. Soil and water may be contaminated and non-target species may be affected. In addition, repeated use of insecticides often leads to the development of resistance in mosquitoes, reducing their effectiveness against target species over time [19]. Insect traps, although more environmentally friendly, have limited effectiveness and require huge densities for mass trapping to be effective [31]. Their costly installation and maintenance make large-scale deployment difficult, and their effectiveness in interrupting the transmission of these viruses remains to be proven [57]. Several genetic control methods also appear to be effective [21], but the unpredictable consequences of the spread of genetically modified mosquitoes in ecosystems raise important ethical and environmental questions and are poorly accepted by the general public.

Given these limitations, there is an urgent need to develop innovative mosquito control methods that are more effective, affordable, environmentally friendly, and acceptable to the public. In this context, the development of alternative control methods, such as the sterile insect technique (SIT), is an emerging and booming technology. SIT is based on the release of irradiated sterile males that sterilize females when mating and thus reduce the target population over time. This species-specific and environmentally friendly autocidal method has a long history of successful large-scale implementation against various insect pests since the 1950s. It is not subject to any regulations relating to genetically modified organisms [8]. Mutations resulting from radiation exposure are inherently random; they are different in each released insect. This randomness limits the potential for the target population to develop resistance, in contrast to the incompatible insect technique (IIT). In fact, IIT-induced sterility depends on the status of the target population and can lead to resistance [62]. SIT and IIT can be combined to avoid this problem. This SIT-IIT combination is effective against *Ae. albopictus* and *Ae. aegypti*: various settings are currently being tested in 39 countries, with a conditional phase-based approach [50]. Singapore has reached the operational level, after demonstrating significant entomological and epidemiological impacts, as well as the cost-effectiveness of the SIT-IIT combination [2], while maintaining communication to obtain public support. However, SIT presents several constraints, including the cost of sterile male mass release, a rapid recovery of target populations when stopping releases, and a sensitivity to invasion of the treated areas by females from neighboring untreated areas.

Recently, we proposed a new strategy, the boosted sterile insect technique (boosted SIT): sterile males are used to disseminate biocides or bio-pesticides by transfer to females during mating and subsequent contamination of breeding sites during oviposition, favored by the skip-oviposition behavior of *Aedes* females [7]. A priori models predicted that boosted SIT, based on two complementary action modes, should be more effective than SIT. On the one hand, sterility is induced in females mating with sterile males (SIT effect). On the other hand, the survival and molting rates of immature aquatic stages are affected by biocides such as pyriproxyfen, which prevent the emergence of adults from larvae (boosted effect) [18, 26, 47]. This strategy combines the benefits of SIT with the auto-dissemination of pyriproxyfen [11, 16]. However, the efficacy of auto-dissemination is variable [54]. It relies on the assumption that the biocide is brought to the breeding sites by contaminated females. Therefore, it should depend on their density, which decreases when the control program is effective, thus reducing its effect. In contrast, SIT is more effective when the target population is decreasing and the ratio of sterile males to fertile males increases consecutively.

A semi-field experiment under semi-controlled conditions was implemented in La Reunion from November 2020 to April 2021, to study (i) the efficacy of two sterile-to-fertile male ratios – 1:1 and 5:1, and (ii) the impact of pyriproxyfen contamination on the suppression of artificial populations of *Ae. albopictus* in cages. The experiment aimed to compare SIT and boosted SIT for these two ratios, and to provide guidelines for the implementation of a field trial using boosted SIT.

## Materials and methods

### Laboratory mosquitoes

The *Ae. albopictus* males and females used in this study originated from eggs collected in the field at Saint-Marie, La Reunion, and have been reared in the laboratory since 2014 in Bugdorm cages (30 × 30 × 30 cm; MegaView, Taichung, Taiwan), in the IRD insectarium located at CYROI Saint-Denis, La Reunion. The rearing procedure was investigated to maintain colony production at a range of 5,000–10,000 adults per week [24].

Laboratory mosquitoes were reared in a climate-controlled insectarium (*T*: 7 ± 2 °C, RH: 75 ± 2%, light: 12L: 12D). The females were blood fed with a Hemotek system (Discovery Workshops, Accrington, United Kingdom), that is, stainless steel plates (10 cm), filled with beef blood and covered with a Parafilm membrane. The eggs hatched in water with the addition of dehydrated rabbit food (hay pellet, Compagnie des Grains du Capricorne, Le Port, La Reunion). They were left in rearing water for 24 h. The larvae were collected and reared at a density of approximately 500 L1 larvae per tray (30 × 40 cm) containing one liter of water. They were fed dry pellets composed of 50% rabbit food and 50% fish food (Sera Koi Food, Sera, Heinsberg, Germany). The first pupa appeared after 5 days of egg hatch. The male and female pupae were separated according to the size of the pupa and a manual check was performed according to the morphology of the terminalia observed with an optical microscope (Leica MZ6 X25). For fertile insects, female and male pupae were allowed to emerge separately. For the production of sterile males, only male pupae were kept for later steps. For each replicate, a total of 1,400 females, 1,100 fertile males, and 3,200 sterile males were used, including the flight test mosquitoes.

Male pupae (range of age 24–30 h) were sterilized in Petri dishes at 40 Gy using the blood sterilization facility (Blood X-RAD 13-19, Cegelec, France) of the *Établissement Français du Sang* located at the University Hospital Center in Saint-Denis, La Reunion [42, 43]. After irradiation, the male pupae were brought back to the laboratory and left to emerge in cups in Bugdorm cages (30 × 30 × 30 cm). After the emergence of adults, the cups were removed and dead pupae and adult mosquitoes were counted.

Two indicators were used to assess the quality of reared mosquitoes: (i) the hatch probability for females, and (ii) the escape probability for boosted sterile males. Their reference values were estimated from control batches (cages without boosted sterile mosquitoes). The quality was considered good if the estimated probabilities of hatch and escape were greater than 0.70.

The escape probability was the probability to fly away from a flight-test device designed for this purpose [13, 39]. After chilling and coating with pyriproxyfen, the boosted sterile males were released into Bugdorm cages with a 10% sugar solution and left to rest for 24 h at 28 °C. Each batch of 100 fertile males, 100 sterile males, and 100 boosted sterile males was then placed in a flight test tube and allowed to escape for 2 h. Finally, the flight tests were transferred to a cold room and the number of dead mosquitoes inside and outside the tubes was counted. The probability of escape was calculated by dividing the number of escaped males by the total number of males. It was used as a proxy for the competitiveness of sterile males.

These indicators were estimated using a Bayesian mixed-effect binomial logistic regression model. They were also estimated for each category *i* of treatment (*i* ≠ ref), allowing the estimation of relative risks *RR*_i,ref_ = *P*_*i*_/*P*_ref_, with *P* the probability of hatch or escape.

### Pyriproxyfen

**Figure B1:**
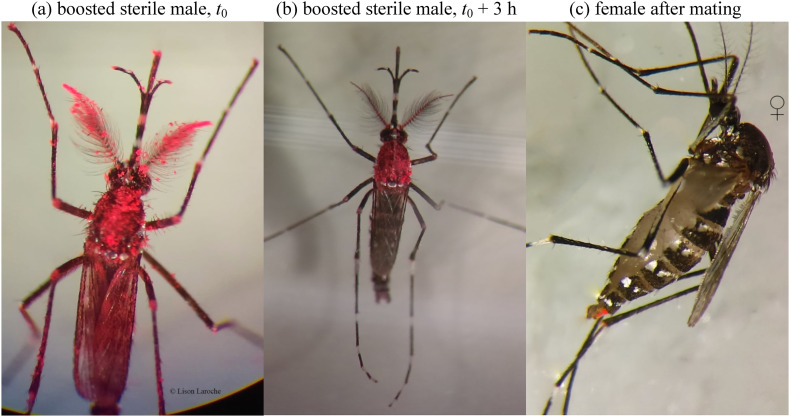
*Aedes albopictus* mosquitoes coated with a powder made of a mixture of pyriproxyfen and a fluorescent dye to preserve the possibility of distinguishing between released boosted sterile males and wild males in field surveys. (a) Boosted sterile male just after impregnation; (b) the same, 3 h later; (c) female after mating with this male: pyriproxyfen powder transferred from boosted sterile male to female during mating can be seen on its genitalia. Photo credit: L. Laroche, UMR Mivegec, Montpellier.

To avoid contamination, pyriproxyfen was handled in a separate building from the insectarium. A formulation of 40% pyriproxyfen and 60% fluorescent powder (pink, DayGlo Color Corp., Cleveland, OH, USA) was used at three different concentrations: 1 mg, 2 mg, and 1,000 mg/200 males. To do this, the formulation was weighed and transferred to 100 mL cylindrical containers. For better adherence of the powder, their inner surface was rubbed with sandpaper. The containers were then shaken and placed in a 4 °C cold room. The sterile males were placed in the 4 °C cold room for 5–10 min (time needed to knock down them), then poured into the containers, which were rotated for 30 s (approximately 25 full rotations) until the mosquitoes were evenly coated [12]. The mosquitoes were then released into semi-field cages for the experiment: males first and then females (Appendix B, Fig. B1). Ten boosted males were sampled in individual 2 mL glass vials for dosage of pyriproxyfen.

The concentration of pyriproxyfen in boosted males was estimated by high performance liquid chromatography (HPLC) analysis at the University of Montpellier, France according to the method described in [33]. Slight changes were made to the protocol concerning the dose of pyriproxyfen in the water samples. Ten milliliter of water samples were collected from oviposition traps. They were lyophilized and sent to the laboratory. Just before analysis, they were dissolved in 1 mL acetonitrile. After centrifugation and dry evaporation, the supernatant was mixed with 100 μL of acetonitrile. Finally, 10 μL of this solution were injected into the HPLC device for analysis.

### Semi-field trial

**Figure A1:**
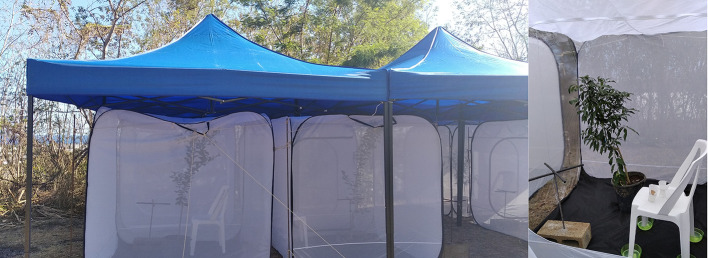
View of the semi-field experiment to assess the efficacy of the boosted sterile insect technique in suppressing populations of *Aedes albopictus*, November 2020 to April 2021, CIRAD La Bretagne Research Station (La Reunion). Left: layout of the cages; right: equipment installed inside the cages.

The experiments were carried out on the campus of the *Centre de Coopération Internationale en Recherche Agronomique pour le Développement* (CIRAD) in Sainte-Clotilde, La Reunion. An overview of the experiment is shown in Appendix A, Figure A1. Semi-field cages (Vermandel, UGS: 80.310, 2 × 2 × 2 m) were placed on gravel and covered with a protective roof from the sun and rain.

The cages were placed at least 20 cm apart, even though pyriproxyfen is only slightly volatile and did not dissipate across the air between cages [52]. Each cage was reused in strict accordance with the same pyriproxyfen condition and sterile-to-fertile male ratio in each replicate to avoid cross-contamination.

An electronic sensor (Thermopuce: Waranet Solutions, Auch, France) was used to measure temperature and relative humidity conditions at the test site between November 2020 and April 2021. Temperatures ranged from 19 to 40 °C, while humidity ranged from 30 to 90%. A peak of 120% humidity was recorded in April 2021 during a rainy spell.

A lemon tree was placed inside each cage to maintain humidity and provide a resting place. A plastic chair placed in talc-filled trays to prevent access by ants and a crossbar allowing feeding with the Hemotek membrane feeding system (Hemotek, Blackburn, UK) were also placed inside each cage. Three 100 mL cups, made of filter paper dipped in a 5% sugar solution and sealed with a cork to prevent adults from drowning, were placed on each chair and changed every 4 days.

Five replicates of 40 days each were conducted between November 2020 and April 2021. The first (replicate 0) was used only to establish a reference situation in which sterile mosquitoes were boosted with a very high dose of pyriproxyfen (1 g/200 sterile males). These data were used as a control for the concentration of pyriproxyfen in the sterile males and its effect on the dosage of pyriproxyfen in the trap water from cages where the sterile males were released.

**Figure 1:**
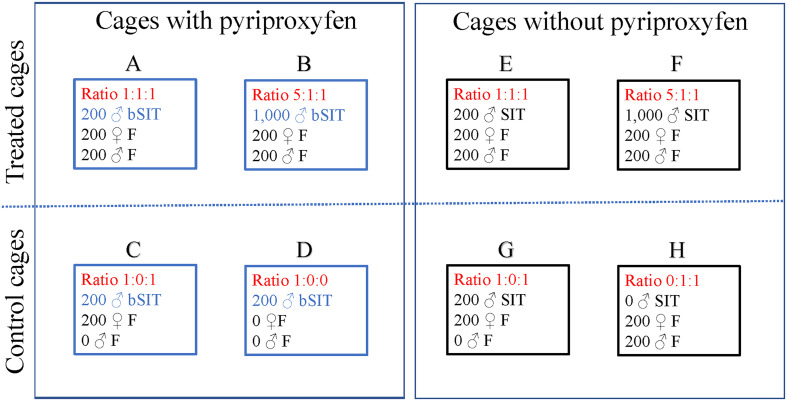
Matrix representation of a replicate of the semi-field experiment implemented at CIRAD La Bretagne Research Station (Sainte-Clotilde, La Reunion), from November 2020 to April 2021, to assess the effect of different settings of the sterile insect technique (SIT) and boosted SIT in suppressing controlled populations of *Aedes albopictus*. The demographic analysis included data from four replicates, with eight mosquito-netted cages by replicate. A fifth replicate was run with a spuriously high amount of pyriproxyfen to boost the sterile males, as a pyriproxyfen positive control. It was not used for the demographic analysis. Fixed proportions, coded *x:y:z*, of adult sterile males (*x*), and fertile males (*y*) and females (*z*) (“F” standing for ‘Fertile” below the code) were released in each cage. Eggs were collected in oviposition traps, counted, dried, and flooded for hatch. Immature stages were monitored until the emergence of adults, or up to 15 days after hatch. Cages A, B, E, and F (upper row of the matrix) were used to mimic the dynamics of immature populations of *Aedes albopictus* under two factors of standard or boosted SIT: (i) sterile-to-fertile male ratio (noted *s*2*f*) of 1:1 (cages A and E), or 5:1 (cages B and F), and (ii) exposure to pyriproxyfen vectored by the sterile males: (cages A and B), or no exposure (cages E and F). The other cages (lower row of the matrix) were used for quality control - hatching of eggs collected from control females; residual fertility in sterile males (cage G), and boosted sterile males (cage C) - capacity of sterile males to vector pyriproxyfen to the breeding sites, with respect to the presence of females (cages C and D).

Eight different conditions were tested in each replicate of the experiment. Adult mosquitoes aged 12 days after hatching were released into 4 cages containing different proportions of females, fertile males, and sterile males only (without pyriproxyfen), and into 4 other cages containing the same proportions of fertile males, females, and boosted sterile males. A description of each condition, including control cages, is given in Figure 1.

**Figure 2:**
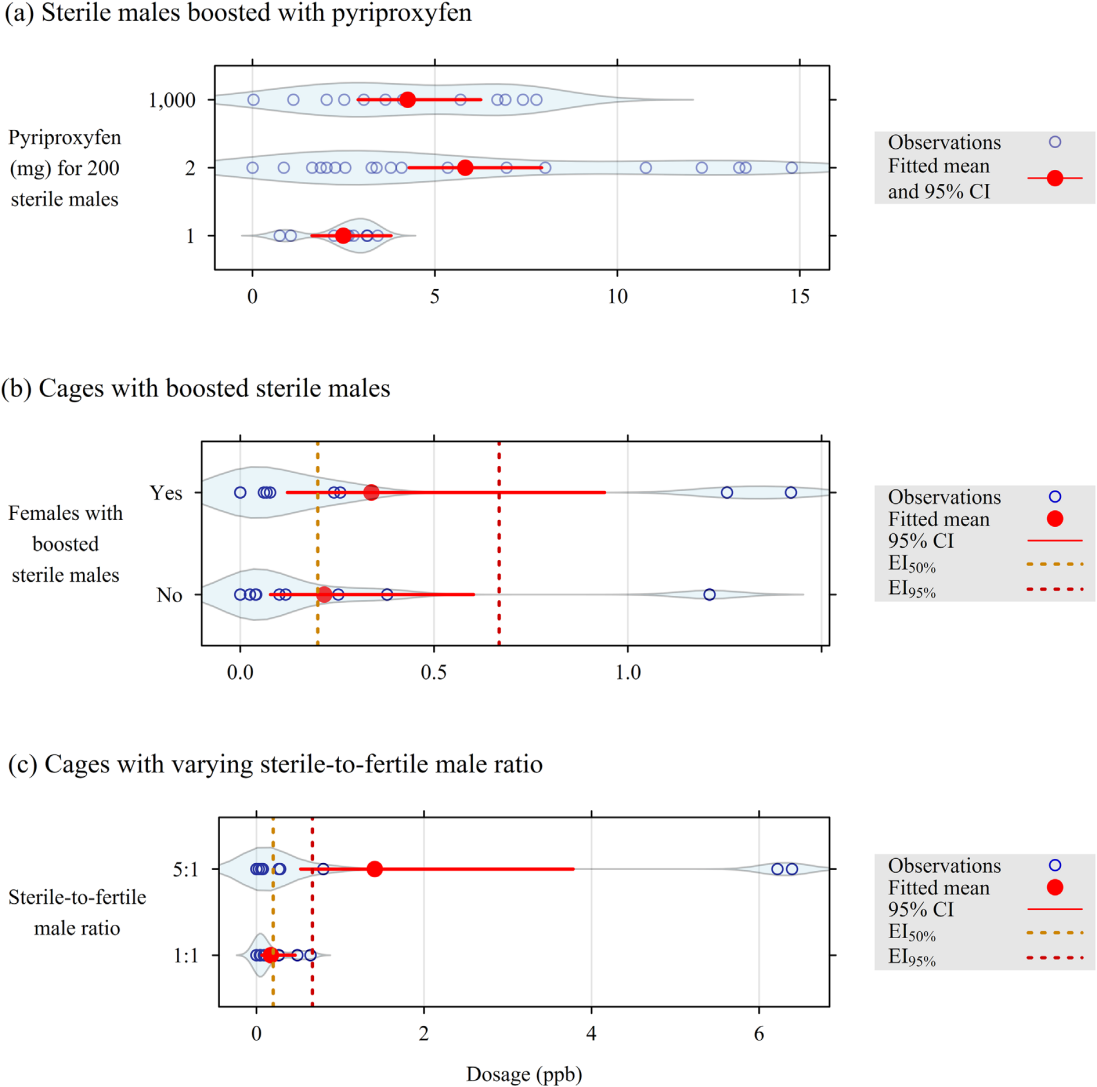
Pyriproxyfen concentrations recorded in a semi-field experiment to assess the efficacy of the boosted sterile insect technique in suppressing populations of *Aedes albopictus*, November 2020 to April 2021, CIRAD La Bretagne Research Station (Sainte-Clotilde, La Reunion). (a) on the boosted males; (b) in the trap water from cages with boosted males, conditionally on the presence of females; (c) in the trap water from cages with females exposed to sterile-to-fertile male ratios of 1:1 and 5:1. EI_50_ and EI_95_: pyriproxyfen concentration (ppb) in water to obtain 50% (*pp* = 0.20 ng/L) and 95% adult emergence inhibition (*pp* = 0.67 ng/L), respectively [47]. The violin plots superimposed to the points are estimates of the probability density of their distribution.

**Figure 3:**
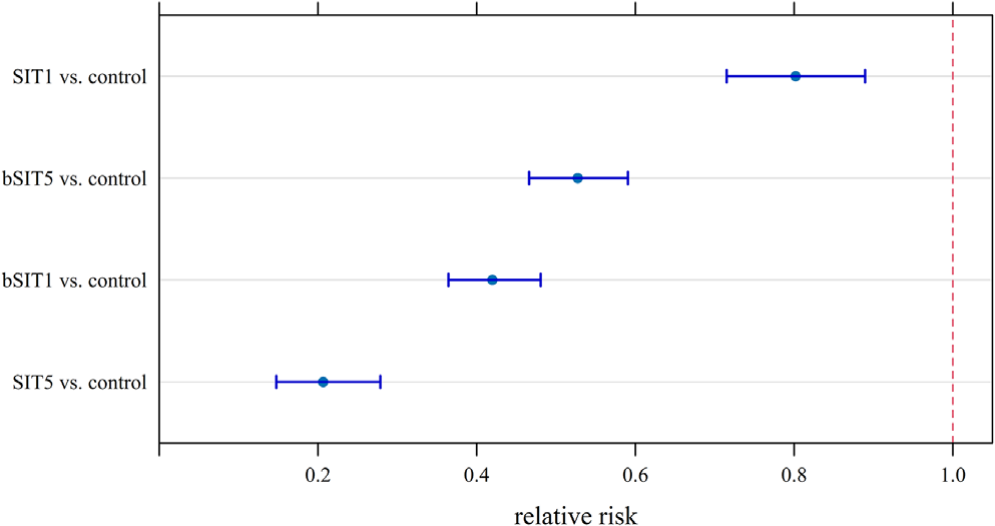
Fitted relative risks of emergence of adult *Aedes albopictus* from eggs exposed to four different options (treatment) of the sterile insect technique (SIT) boosted with pyriproxyfen or not, during a semi-field experiment implemented at CIRAD La Bretagne Research Station (Sainte-Clotilde, La Reunion) from November 2020 to April 2021. Treatment labels: SIT1 or SIT5 with a sterile-to-fertile male ratio of 1:1 or 5:1; bSIT1 or bSIT5: the same with sterile males boosted with pyriproxyfen. Rows were ordered from the most (top) to the least (bottom) effective treatment category against *Aedes albopictus*.

The mosquitoes were left to rest for one day after release to acclimate and mate. On the second day, the females were given a blood meal using the Hemotek membrane feeding system consisting of three stainless steel plates (15 cm in diameter) covered with a Parafilm membrane (Bemis North America, Neenah, WI, USA) and filled with beef blood. They were placed inside the cage for 45 min in the morning and 30 min in the afternoon for three consecutive days.

Two oviposition traps, consisting of a strip of blotting paper (oviposition support) dipped in black pots filled with 250 mL of tap water, were placed after the second day of feeding (third day). These traps were the only breeding sites available to female mosquitoes in the cages. Five days after (the eighth day), the oviposition traps were removed. Adult mosquitoes were collected with a vacuum aspirator and killed. A 10 mL sample was taken from the water in each oviposition trap with a single use pipette, stored in a 30 mL glass vial at −20 °C and lyophilized before analysis. The blotting paper strips were harvested and dried at 27 °C in aluminium foil [61]. The egg papers were returned in aluminium foil, and after 1 week of drying, the hatched and unhatched eggs were counted under a stereomicroscope (natural hatching rate). A sample of 100 eggs was again put in the water of the oviposition traps in 250 mL glass beakers with food consisting of dry pellets (1 mL of 7.5% food for 30 larvae). The eggs were allowed to hatch for 1 day, and the papers were removed and then dried at ambient temperature for 1 day. The hatched and unhatched eggs were counted under a stereomicroscope to estimate the final hatching rate. The immature stages and emerging adults were counted every two days for 15 days and the data were entered and stored in a database.

### Data analysis

The pyriproxyfen concentration was analyzed with generalized linear models assuming a zero-augmented gamma distribution for the response and setting a logarithmic link between the expected response and the linear predictor. Likelihood ratio tests were used to assess the statistical significance of the reduction in deviance associated with the addition of a covariate to the initial model. Wald tests were used to compare the coefficients of a given model with reference values.

For demographic analysis, the statistical unit *i* (*i* = 1, …, 20) was the cage by replicate, *i.e.*, we aggregated the demographic data by cage and replicate, treatment category (Fig. 1) and development stage (larva, pupa, and emerging adult). Three indicators were estimated: (i) hatch probability, (ii) competitiveness of sterile males, and (iii) probability of emergence of adults from the initial number of eggs, for each category of treatment:

- control: no treatment,
- sterile insect technique (SIT) with the sterile-to-fertile male ratio (not irradiated) *s2f* = 1:1 (SIT1),
- SIT with *s2f* = 5:1 (SIT5),
- boosted SIT with *s2f* = 1:1 (bSIT1),
- boosted SIT with *s2f* = 5:1 (bSIT5).

The main features of these data were:

- Demographic events (death, molting) occurring in the successive immature stages acknowledged in this experiment: eggs, larvae, pupae, and adults.
- Longitudinal data: cohorts of eggs were flooded at the same time, and monitored until the emergence of adults, up to 15 days after their hatch. Consecutively, data were clustered and right-censored for mosquitoes surviving at the end of monitoring.
- Lastly, mosquitoes were monitored at fixed time intervals. For those individuals that died or molted to the next stage, we did not know the event date: we only knew it occurred between the former and the current examination.

To address these features, we used the methodological framework defined in Lesnoff *et al.*, 2014 [36], *i.e.*, the analysis of discrete-time survival data, corrected for demographic interactions: competing risks of molting and death.

**Figure C1:**
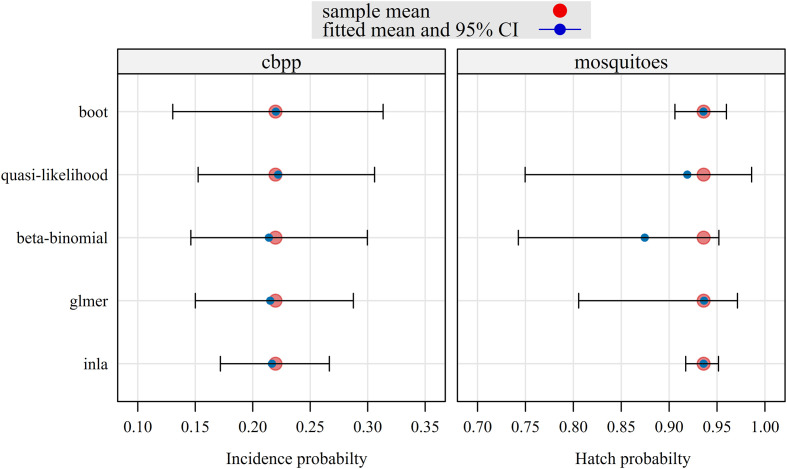
Comparison of probabilities fitted with four logistic regression models. The “boot” model was non-parametric bootstrap (resampling with replacement), used as a gold standard. First set of rows (cbpp): serological incidence probability of contagious bovine pleuropneumonia in newly infected cattle herds from Ethiopian Highlands (survey data collected in early 2000s). Second set of rows (mosquitoes): hatch probability estimated for *Aedes albopictus* eggs (control group) collected during a semi-field experiment in La Reunion (2021). *P*_obs_ observed probability; *P*_fit_ fitted probability; LL lower 95% credible limit; UL upper 95% credible limit.

An important aspect of these data was the frequent occurrence of mosquito cohorts for which the response was perfectly or nearly separated by the treatment category (Appendix C, Table C1). For instance, no eggs hatched in the SIT5 cohort with row index R13, while nearly all of them hatched in cohorts from the control group (*e.g.*, row index R17: 215/228). When such observations were fitted with maximum-likelihood methods, their log-likelihood became infinite or undefined (log of the product of values close, or equal to 0 or 1). Model coefficients and their variance-covariance matrix were biased. This so-called Hauck-Donner effect [28] may have severe consequences for the conclusions drawn from data analysis [23]. We were confronted with this problem, as described in Appendix C, Tables C2–C3 and Figure C1. In practice, we recommend to carefully check differences between observed and fitted data, and/or spuriously high estimates of coefficients standard error.

To estimate the hatch probability (*P*_h_) and the probability of adult emergence from larvae (*P*_a_), we used a Bayesian mixed-effect binomial logistic regression model. The treatment was the single fixed effect included in the model. We also defined an observation-level random effect associated with the model intercept [27].

The estimate of *P*_*a*_ was the count of emerging adults *n*_meta_, reported to the initial number of larvae *e*_meta_. This count was corrected for the demographic interference between the molting and death processes (death counts *l*_death_ for larvae and *n*_death_ for pupae) [36]:

$$P_{a}=\frac{n_{\mathrm{meta}}}{e_{\mathrm{meta}}-\frac{l_{\mathrm{death}}+n_{\mathrm{death}}}{2}}.$$

In both cases (*P*_*h*_ and *P*_*a*_), 10,000 simulated fitted values were drawn in the posterior distribution of the corresponding model coefficients and stored in matrices *M*_*h*_ and *M*_*a*_, both with 20 rows (5 treatment categories × 4 replicates), and 10,000 columns (one for each simulated dataset). Rows were aggregated by treatment category. Estimates of *P*_*h*_ and *P*_*a*_, and their lower and upper 95% credible limits were obtained taking the mean and quantiles 2.5% and 97.5% of the aggregated rows.

The matrix *M*_*h*_ was also used to estimate the competitiveness (*Cp*) of sterile males, *i.e.*, their capacity to mate with females in the presence of fertile males, for each treatment category (but control) *i* [9, 22] with *s*2*f* the ratio between sterile and fertile males:

$$Cp_{i}=\frac{P_{h,\mathrm{control}}-P_{h,i}}{P_{h,i}}\times \frac{1}{s2f}.$$

The probability *P*_*e*_ of adults emerging from eggs was estimated from the arithmetic product

$$M_{e}=M_{h}\times M_{a}.$$

The matrix *M*_*e*_ was used to estimate the relative risks of emergence with respect to controls

$$RR_{i,\mathrm{control}}=P_{e,i}/P_{e,\mathrm{control}}$$

as well as their 95% CI.

In this study, *α* was set to 0.05. Data analysis, including plots and tables, was performed with R [48], a software environment for statistical computing and graphics. All the datasets and R code are available on reasonable request from the second author of this paper. The manuscript was prepared with R add-in packages rmarkdown [59] and bookdown [58], and the MS Word document was compiled with pandoc [32].

## Results

### Quality of laboratory mosquitoes

**Figure B2:**
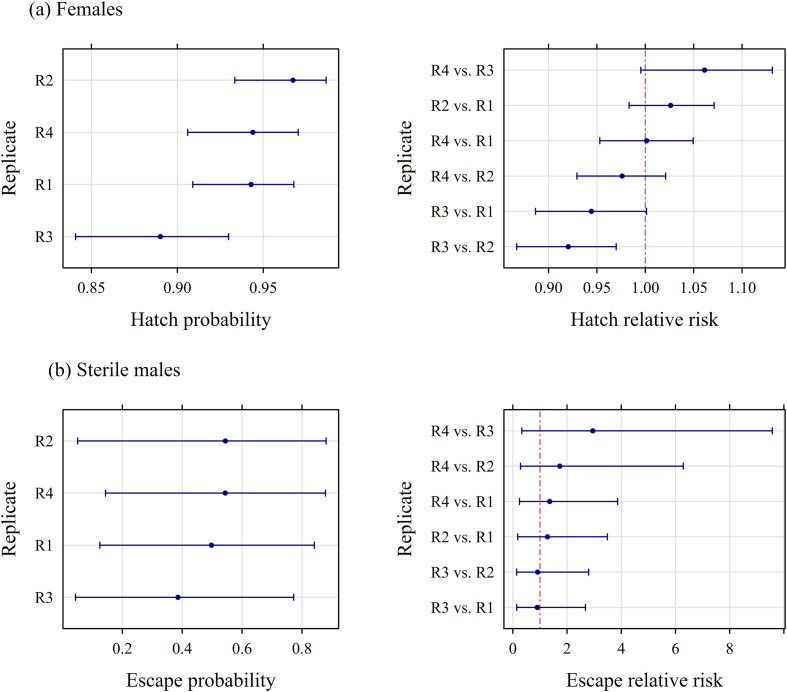
Quality of mosquitoes used during a semi-field experiment to assess the effect of pyriproxyfen on the development of the immature stages of *Aedes albopictus*, CIRAD La Bretagne Research Station (La Reunion), November 2020 to April 2021. (a) Females (hatch probability and relative hatch risk); (b) sterile males (escape probability and relative risk of escape). The labels on the *y-*axis of each plot stand for “Replicate *x*” (*x* = 1, …, 4).

In the control groups, the hatch probability was above 0.70 in all replicates (Appendix B, Fig. B2a). In sterile males from the control group, the escape probability was below the recommended 0.70 threshold, but the difference with this threshold was not significant (Appendix B, Fig. B2b, *α* = 0.05). However, the escape probability for the third replicate was significantly lower than for the other replicates. This was probably due to the fact that the mosquitoes used for the flight test were mistakenly not sugar-fed after being boosted with pyriproxyfen. However, the mosquitoes released into the field cages were properly fed and were therefore not affected by this poor quality.

**Figure B3:**
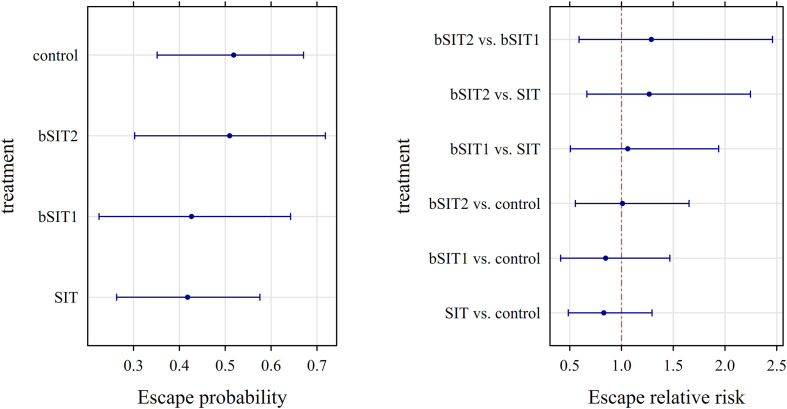
Escape rate and relative risk of escape for sterile males used for a semi-field experiment implemented from November 2020 to April 2021 at CIRAD La Bretagne Research Station (La Reunion), to assess the effect of pyriproxyfen on the development of the immature stages of *Aedes albopictus*. Treatment labels: control: fertile males, no pyriproxyfen; SIT: irradiated males no pyriproxyfen; bSIT1: boosted irradiated males, 1 mg pyriproxyfen/200 mosquitoes; bSIT2: boosted irradiated males, 2 mg pyriproxyfen/200 mosquitoes.

The results by treatment category are shown in Appendix B, Figure B3. The escape probability of the sterile males decreased with respect to the control mosquitoes, but this decrease was not significant.

### Pyriproxyfen

In boosted males, the concentration of pyriproxyfen did not vary consistently with the amount of pyriproxyfen used to coat them (Fig. 2a):

- the difference in pyriproxyfen concentration between sterile males boosted with a powder containing 1 or 1,000 mg pyriproxyfen/200 males was not significantly higher than zero (Wald test, *t* = 1.84, *p* = 0.074),
- in contrast, the difference in pyriproxyfen concentration between sterile males boosted with a powder containing 1 or 2 mg pyriproxyfen/200 males was significantly higher than zero (Wald test, *t* = 3.20, *p* = 0.003), but with a higher variance for the 2 mg group.

We selected the dose of 2 mg/200 sterile males for two of the four replicates, resulting in measured doses ranging from 0.003 to 14.780 ng of pyriproxyfen per sterile male. Furthermore, in cages containing boosted males and no fertile males (Fig. 2b), the concentration of pyriproxyfen in the trap water did not change, regardless of whether the females were released into the same cages (likelihood ratio test – LRT: *χ*^2^ = 0.99, *df* = 1, *p* = 0.547).

Finally, increasing the ratio between boosted and fertile males from one to five – with a constant number of females – resulted in a higher concentration of pyriproxyfen in the trap water (Fig. 2c): LRT *χ*^2^ = 18.8, *df* = 1, *p* = 0.006). In this sample, this difference was related to two large outliers (concentration > 6 ppb), likely caused by dead-boosted males falling into the water.

These last two observations (Figs. 2b and 2c) provided clear evidence that boosted males can effectively vector pyriproxyfen directly to the breeding sites.

### Efficacy assessment

The hatch probability, as well as the competitiveness of sterile males, is shown in Table 1. As expected, eggs from the control group had the highest hatch probability: *P*_h,fit_ = 0.936 95% CI [0.917; 0.952]. The lowest hatch probability was observed for SIT5: *P*_h,fit_ = 0.248 [0.220; 0.275]. Sterile males in this category also had the best competitiveness: *Cp* = 0.562 [0.491; 0.663]. The eggs in each boosted category had a higher hatch probability rate than those in the corresponding non-boosted category:

- The efficacy loss was small and not significant for bSIT1 *vs*. SIT1: *δ* = 0.009: simulation test, H_0_: *δ* = 0, H_1_: *δ* < 0, *B* = 10^4^, *p* = 0.3554.
- In contrast, it was large and significant for bSIT5 *vs*. SIT5: *δ* = 0.3229, simulation test, H_0_: *δ* = 0, H_1_: *δ* < 0, *B* = 10^4^, *p* < 10^4^.

**Table 1:** Hatch probability and competitiveness of *Aedes albopictus* eggs and adult males exposed to different options (treatments) of the sterile insect technique (SIT) boosted with pyriproxyfen during a semi-field experiment at CIRAD La Bretagne Research Station (Sainte-Clotilde, La Reunion) from November 2020 to April 2021. *P*_h,obs_ observed probability; P_h,fit_ fitted probability; LL lower 95% credible limit; UL upper 95% credible limit; Cp competitiveness of sterile males. Treatment labels: “control” fertile males (no treatment); “SIT1”, “SIT5” SIT with a sterile-to-fertile male ratio 1:1 or 5:1; “bSIT1”, “bSIT5” the same with sterile males boosted with pyriproxyfen. Rows were ordered from the most (top) to the least (bottom) effective treatment to prevent the hatch of eggs.

Treatment	*P*_h*,*obs_	*P*_h*,*fit_	LL	UL	*Cp*	LL	UL
SIT5	0.246	0.248	0.220	0.275	0.562	0.491	0.633
bSIT5	0.576	0.577	0.545	0.609	0.125	0.112	0.137
SIT1	0.762	0.764	0.720	0.802	0.229	0.186	0.274
bSIT1	0.773	0.773	0.741	0.803	0.211	0.185	0.237
Control	0.936	0.936	0.917	0.952			

The lowest emergence probability of adults from eggs was observed for the SIT5 treatment category (Table 2): *P*_e,fit_ = 0.132 [0.095; 0.176]. Interestingly, this probability was similar for bSIT1 (*P*_e,fit_ = 0.267 [0.234; 0.301]) and bSIT5 (*P*_e,fit_ = 0.335 [0.301; 0.370]).

**Table 2:** Emergence probability of adult *Aedes albopictus* from eggs exposed to four options (treatments) of the sterile insect technique (SIT) boosted with pyriproxyfen during a semi-field experiment at CIRAD La Bretagne Research Station (Sainte-Clotilde, La Reunion) from November 2020 to April 2021. *P*_e,fit_ fitted probability; LL lower 95% credible limit; UL upper 95% credible limit. Treatment labels: “control” fertile males (no treatment); “SIT1”, “SIT5” SIT with a sterile-to-fertile male ratio 1:1 or 5:1; “bSIT1”, “bSIT5” the same with sterile males boosted with pyriproxyfen. Rows were ordered from the most (top) to the least (bottom) effective treatment prevent the emergence of adults.

Treatment	*P*_e*,*fit_	LL	UL
SIT5	0.132	0.095	0.176
bSIT1	0.267	0.234	0.301
bSIT5	0.335	0.301	0.370
SIT1	0.509	0.461	0.555
Control	0.636	0.602	0.670

The relative risks of emergence of adults from eggs (Fig. 3) summarize the main conclusions of the demographic study. All the treatments allowed a large and significant reduction of the adult emergence probability, with respect to (w.r.t.) the control group:

- The most effective strategy was SIT5 with a fitted *RR* = 0.207 [0.149; 0.279], *i.e.*, a 5-fold (or 80%) reduction w.r.t. the control group.
- The SIT at a ratio of 1:1 was the least effective in suppressing semi-field populations of *Ae. albopictus*: *RR* = 0.802 [0.715; 0.887], *i.e.*, a 20% reduction of adult emergence w.r.t. the control group.
- The boosted SIT was not as effective as SIT for each sterile-to-wild male ratio 1:1 and 5:1. However, similar reductions in the probability of adult emergence were obtained with bSIT1 (*RR* = 0.419 [0.364; 0.478]) and bSIT5 (*RR* = 0.527 [0.467; 0.591]). Therefore, boosted SIT may remain effective for suppressing *Ae. albopictus* populations both at the start of a mosquito control program (*i.e.*, when the density of wild mosquitoes is poorly known), or when the program applies at the seasonal peak of *Aedes* population dynamics.

## Discussion

We conducted a semi-field trial to compare the efficiency of SIT and boosted SIT with sterile-to-wild male ratios of 1:1 and 5:1, and to prepare for a field trial against *Ae. albopictus* in Spain and *Ae. aegypti* in La Reunion [5]. The experimental design probably minimized the potential of boosted SIT, because of poor adhesion and transfer of the pyriproxyfen formulation. Previous research conducted on the *Glossina* model confirmed the importance of developing and evaluating pyriproxyfen formulations best suited for entomovectoring [33]. Also, the semi-field trial mimics a closed environment where females laying fertile eggs cannot migrate from surrounding populations, a phenomenon that is generally observed in open field trials [4, 10, 53]. Our controlled conditions thus represented an ideal SIT scenario applied against an isolated target population. Under such conditions, the most effective strategy was SIT in a sterile-to-fertile male ratio of 5:1, considering the probability that adults emerge from eggs exposed to four treatment options. This result was mainly related to a stronger reduction in the hatch probability in this ratio than any other option (Fig. 3). This is in line with general recommendations to use the SIT at a ratio of 5:1 to 10:1 [24].

Boosted SIT with sterile-to-wild male ratios of 1:1 and 5:1 both provided a reduction in adult emergence by nearly 50%. This result is not consistent with the higher concentration of pyriproxyfen in a ratio of 5:1 between boosted and fertile males (Fig. 2c). However, this higher concentration was caused by two outliers: otherwise, the concentrations were similar in the two ratios for the other samples. We consider these outliers to correspond to boosted males falling into the ovitraps, as observed in Kentucky [40] and Spain [5]. Furthermore, two reasons might explain the variations in pyriproxyfen concentration and the low values sometimes found in the trap water.

- Firstly, evaporation of the water from the trap in the cages required adjustment of the water levels in some traps to allow the females to lay their eggs. Such adjustments were similar to the succession of evaporation and rainfall in field breeding sites, with the same consequent effect on pyriproxyfen concentration.
- Second, the detection of pyriproxyfen in some control cages could be related to the dry-freezing process: this lasted more than 10 h, with the risk of cross-contamination between samples due to volatile particles emitted during the process [15].

The pyriproxyfen coating process slightly reduced the escape probability of boosted sterile males, and more importantly their competitiveness, especially in the bSIT5 treatment (Table 1 and Appendix B, Fig. B3). At this dose, even the fluorescent powder alone reduced the quality and survival of sterile males in a previous experiment, so we can hypothesize that this effect is mainly caused by physical action of the particles at high concentration, rather than a toxic effect of pyriproxyfen or the impact of the chilling and handling processes. As a matter of fact, the quality of males was not reduced at a lower dose, although these processes remained unchanged [13]. However, this lower quality did not severely hamper the efficiency of the boosted SIT in reducing the emergence probability of adults (Table 2 and Fig. 3). This is in line with the good suppression rate observed in a field trial performed in La Reunion with the same formulation despite a low ratio between sterile and wild males: 0.1 for the first 2 months of the experiment, and 11.2 for the remainder of the period (mean: 0.7) [5].

We did not observe any significant effect of increasing the dose applied to sterile males on the residual amount measured on their surface, as if the boosted males were saturated by a certain amount of powder. It might be related to electrostatic charges on the surface of the mosquitoes or to the cluster they sometimes formed when intermingling inside the pot. During the impregnation of the sterile males, we observed that with 1 mg/200 sterile males, the powder was completely adsorbed by the mosquitoes and the rough walls of the jar; therefore, we suspect that this amount was not sufficient to saturate all the mosquitoes. With 2 mg/200 sterile males, the powder seemed evenly distributed throughout the mosquito cluster. With 1 g/200 sterile males, the males on the outer side of the cluster were quickly saturated with the powder, preventing the mosquitoes in the inner side from receiving pyriproxyfen.

The dose of 2 mg/200 sterile males led to concentrations of pyriproxyfen in the trap water below the emergence inhibition rate of 95% (EI_95%_), except for two outliers (see above). This shows that the pyriproxyfen formulation that was used in this trial (a mixture of fluorescent dye and pyriproxyfen) was not optimal in this semi-field experiment. Despite the use of this sub-optimal formulation during the field trials in Spain and Reunion, boosted SIT still led to strong suppression (>90%) of *Ae. albopictus* and *Ae. aegypti*, respectively [5]. This suggests considerable potential to improve larval habitat contamination, as also found for another formulation used in a field trial in China using *Wolbachia*-infected males as vectors for pyriproxyfen [37]. Developing a more efficient pyriproxyfen formulation is a priority to ensure the scalability of boosted SIT in real-world applications.

The known effects of pyriproxyfen on *Ae. albopictus* are suppression of egg production and hatching, as well as inhibition of growth at the immature stage [30, 45]. The most important finding in this study was that the females were weakly involved in the dissemination of pyriproxyfen to breeding sites. The original concept of boosted SIT [7] was to use females to contaminate breeding sites after they were contaminated by boosted sterile males. This would lead to a density-dependent contamination of larval habitats, which would decrease with female density, as observed in the auto-dissemination technique [17]. In the boosted SIT models [18, 26, 47], this reduction in larval sites contamination by pyriproxyfen was compensated by an increase in the sterile-to-fertile male ratio, leading to increased induced sterility, as observed in SIT. In these simulations, most of the reduction effect was related to pyriproxyfen, whereas the impact of SIT became predominant when the target population was reduced. These model predictions may change when considering direct contamination of larval habitats by boosted males.

Our results suggest that after initial suppression of the target population by boosted SIT, which can be obtained with a low sterile-to-fertile male ratio, it is possible to switch to standard SIT, without reducing the efficiency of the control program, once this ratio reaches 5:1. This will prevent the development of resistance to pyriproxyfen in the target population, while reducing the risk of non-target effects. Importantly, exposure to sublethal doses may lead to the development of resistance to this compound or even to cross-resistance to other insecticides.

## Conclusion

By providing a way to directly and specifically contaminate larval habitats through sterile males, in a manner that does not depend on the density of the target population, boosted SIT has the potential to revolutionize the control of *Aedes* mosquitoes, especially if we can use specific bio-pesticides such as densoviruses [6, 46] and create formulations that improve the transfer of pyriproxyfen from sterile males to larval habitats.

## Appendix C. Hauck-Donner effect with binomial logistic regression models of hatch probability

### C1: Demographic data of *Aedes albopictus* immature stages

This dataset (Appendix C, Table C1) was built from demographic data collected during the monitoring of immature stages to estimate the probability of adult emergence from *Ae. albopictus* eggs. Each row describes the demographic events recorded for a given cohort of mosquitoes monitored from egg flooding (“e_ini” is the size of the initial egg batch) until the emergence of adults, censored at 15 days post flooding.

**Table C1:** Aggregated demographic data on the immature stages of *Aedes albopictus* collected during a semi-field experiment implemented on CIRAD La Bretagne Research Station (Sainte-Clotilde, La Reunion) from November 2020 to April 2021. Column headers: “idx” row index; “repl” replicate; “cage” cage id; “treatment’: SIT1 or SIT5 sterile males released in cages with a sterile-to-fertile male ratio of 1:1 or 5:1, bSIT1 or bSIT5 the same with sterile males boosted with pyriproxyfen; “e_ini” number of flooded eggs; “e_meta” hatch number; “l_meta” number of larvae molting into pupae; “l_death” number of dead larvae; “n_meta” number of emerging adults; “n_death” number of dead larvae; “ncorr” initial number of larvae corrected with larval and nymphal death (see Materials and methods section).

idx	repl	cage	treatment	e_ini	e_meta	l_meta	l_death	n_meta	n_death	ncorr
R01	1	A	bSIT1	219	169	72	20	62	5	157
R05	1	B	bSIT5	217	121	101	13	75	4	113
R09	1	E	SIT1	50	30	26	4	25	1	28
R13	1	F	SIT5	90	0	0	0	0	0	0
R17	1	H	control	228	215	129	30	113	0	200
R02	2	A	bSIT1	102	80	29	51	1	28	41
R06	2	B	bSIT5	229	210	132	77	35	91	126
R10	2	E	SIT1	130	114	86	19	29	55	77
R14	2	F	SIT5	615	204	16	84	0	13	156
R18	2	H	control	181	175	148	13	64	77	130
R03	3	A	bSIT1	197	135	66	56	56	4	105
R07	3	B	bSIT5	196	81	72	9	60	12	71
R11	3	E	SIT1	209	130	83	47	77	6	104
R15	3	F	SIT5	197	16	16	0	13	3	15
R19	3	H	control	183	163	134	25	120	9	146
R04	4	A	bSIT1	193	164	55	79	51	1	124
R08	4	B	bSIT5	151	63	28	31	24	3	46
R12	4	E	SIT1	117	111	55	56	51	3	82
R16	4	F	SIT5	107	61	42	19	42	0	52
R20	4	H	control	177	167	141	10	135	1	162

#### Uncovering the Hauck-Donner effect (HDE)

We attempted to highlight this effect on the estimated hatch probability. According to the literature, should this effect occur, we would expect to find downward biased estimates, with spuriously high variance and wide 95% credible intervals compared with unbiased estimates provided by a gold standard method [23, 28, 60].

We chose the hatch probability in the control group, because its estimate was expected to be close to one in the control group [44, 56, 61]. Therefore, these data were exposed to the risk of HDE.

We used four logistic regression models suitable for the analysis of overdispersed proportions [25, 27, 29, 49] (Appendix C, Table C2):

- two marginal models, i.e., only focusing on an estimate of population means, fitted with functions from the R add-in package aods3 for the analysis of overdispersed data [35]:
- “aodql”: quasi-likelihood model with the quasi-binomial distribution family (“qbin”),
- “aodml”: beta-binomial model with the beta-binomial distribution family (“bb’).

**Table C2:** Logistic regression models used to estimate the hatch probability of *Aedes albopictus* eggs collected during a semi-field experiment implemented on CIRAD La Bretagne Research Station (Sainte-Clotilde, La Reunion) from November 2020 to April 2021.

Model	Type	Package	Reference	Functions	Simulation
quasi-likelihood	marginal	aods3	[35]	aodql	Monte Carlo
beta-binomial	marginal	aods3	[35]	aodml	Monte Carlo
binomial, frequentist	random effect	lme4	[3]	glmer	Parametric bootstrap
binomial, Bayesian	random effect	INLA	[51]	inla	Posterior samples

**Table C3:** Comparison of probabilities fitted with four logistic regression models. The “boot” model was non-parametric bootstrap (resampling with replacement), used as a gold standard. First set of rows (cbpp): serological incidence probability of contagious bovine pleuropneumonia in newly infected cattle herds from Ethiopian Highlands (survey data collected in early 2000s). Second set of rows (mosquitoes): hatch probability estimated for *Aedes albopictus* eggs (control group) collected during a semi-field experiment in La Reunion (2021). *P*_obs_ observed probability; *P*_fit_ fitted probability; LL lower 95% credible limit; UL upper 95% credible limit.

Dataset	Model	*P*_obs_	*P*_fit_	LL	UL
Cbpp	boot	0.220	0.220	0.130	0.313
	quasi-likelihood	0.220	0.222	0.153	0.306
	beta-binomial	0.220	0.214	0.146	0.300
	glmer	0.220	0.215	0.150	0.288
	inla	0.220	0.217	0.172	0.267
Mosquitoes	boot	0.936	0.936	0.906	0.960
	quasi-likelihood	0.936	0.919	0.750	0.986
	beta-binomial	0.936	0.875	0.743	0.952
	glmer	0.936	0.936	0.806	0.971
	inla	0.936	0.936	0.917	0.952

In both cases, a logit link was set between the expected mean *μ* and the linear predictor *η*:

$$\log\left(\mu /\left(1-\mu \right)\right)=\eta =b_{0}+b_{1}\times x_{i}.$$

The model intercept *b*_0_ was the expected mean for the control group on the link scale; *b*_i_ was the signed difference (on the link scale) between the means of the treatment category *i*, and of the control group; *x*_*i*_ was a dummy variable coded 1 for the category of treatment *i* and 0 elsewhere.

- two mixed-effect models with the same fixed effect as for the marginal models, and a random effect *u*_j_ to account for observation-level random fluctuations around the population means. For observation *j*, the linear predictor was thus:

$$\log\left(\mu_{j}/\left(1-\mu_{j}\right)\right)=\eta_{j}=b_{0}^{*}+b_{1}^{*}\times x_{i}+u_{j}$$

with $b_{0}^{*}$, $b_{1}^{*}$ the fixed-effect coefficients estimated conditionally on the random effect *u*_j_, which was:

- either a random variable assumed to follow a Gaussian distribution of mean 0, and variance *σ*^2^ in the frequentist mixed-effect model “glmer” fitted with a first-order Laplace approximation of the likelihood [3],
- or a hyper-parameter with a prior log-Gamma distribution and precision (1/variance) = 5 × 10^−5^ in the Bayesian mixed-effect model “inla” fitted with an integrated nested Laplace approximation of the Markov chain process [51].

Credible intervals for the estimated probabilities were obtained from simulations of the fitted values according to the estimated model coefficients [14, 41]:

- for the marginal models: Monte Carlo simulations assuming a multivariate Gaussian distribution of model coefficients [55];
- for the frequentist random effect models: parametric bootstrap, that is, resampling model residuals with replacement;
- for the Bayesian models: sampling the posterior distribution of model coefficients.

In addition, hatch probability and its credible interval were estimated with a non-parametric bootstrap procedure (resampling the data with replacement), used as a gold standard in this comparison (internal control).

Each model was fitted with two datasets: (i) mosquitoes: the demographic data for *A. albopictus* presented above (Appendix C, Table C1), and (ii) cbpp: a counterfactual dataset (external control) describing the serological incidence of a cattle disease: contagious bovine pleuropneumonia (cbpp) in newly infected herds from the Ethiopian highlands. This dataset is available in the R add-in package “lme4” [3]. The second author of this paper participated in the survey design, field work, and data analysis. No sign of HDE was detected with the usual logistic regression models [34].

The results of model comparisons are shown in Appendix C, Table C3 and Figure C1.

With the cbpp dataset, all the models provided unbiased mean estimates and similar credible intervals.

With the mosquitoes dataset, the two marginal models (“quasi-likelihood” and “beta-binomial”) provided downward biased estimates of hatch probability, as well as spuriously wide 95% credible intervals. For these models, we had a strong suspicion for the occurrence of an HDE.

In addition, the frequentist “glmer” model provided much wider credible intervals than the Bayesian “inla” model. The latter was the only one among the four compared models to provide estimated mean and variance in line with the non-parametric bootstrap method “boot”.

For the revised version of this manuscript, we changed our estimation method from “beta-binomial” and “glmer” models (used in the initial version), to “inla” models. Fortunately, the main trends and conclusions did not change. Nevertheless, some partial conclusions were reversed, such as the effect of SIT1 for which we did not reject the null hypothesis of no effect on the probability of adult emergence in the first place, while we could reject it with the HDE-free results.
